# Hyposkillia and Critical Thinking: Lost Skills of Doctors

**DOI:** 10.21699/ajcr.v1i1.9

**Published:** 2010-08-14

**Authors:** Tayyaba Batool

**Affiliations:** Department of Paediatric Surgery, National Institute of Child Health Karachi, Pakistan

**Dear Sir**

Imperforate hymen is the most frequent obstructive anomaly of the female genital tract. It can be diagnosed on prenatal ultrasound as bladder outlet obstruction, due to hydrocolpos or mucocolpos [1] and postnatally on physical examination of the perineum and abdomen. No additional tests or investigative tools are required for diagnosis per se. We share our experience of two index cases of imperforate hymen where clinical diagnosis was missed.

**Case 1:** A 21-day-old female baby was presented with complaint of urinary retention. Since bladder was palpable on examination, catheterization was done by the nursing staff on advice of a resident. Urinalysis and ultrasound were advised which turned out to be insignificant. On 5th day of admission it was noticed that despite catheterization lower abdomen was still distended. Perineal examination done at that time revealed imperforate hymen (Fig. 1).

**Figure:**
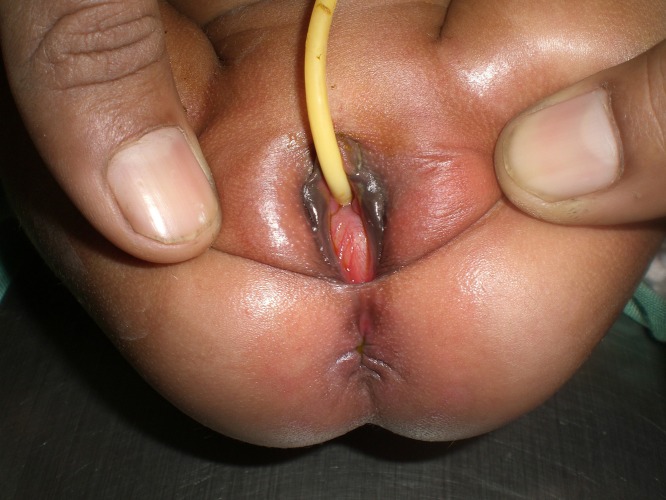
Figure 1: A neonate with imperforate hymen - An obvious clinical finding not to be missed.

**Case 2:** Twelve years old female presented with lower abdominal mass. She was catheterized elsewhere for urinary retention but mass persisted. She was admitted and CT scan abdomen advised which picked up a cystic mass in pelvis (Fig. 2). Surgery planned and on operation table perineal examination revealed an obvious bulge at vaginal orifice. Hymenotomy was performed in both the cases.

**Figure:**
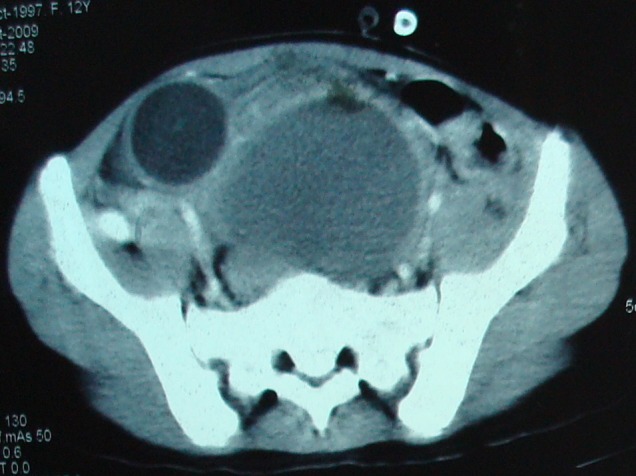
Figure 2: CT scan showing distended vagina and displaced urinary bladder.

Critical thinking is about how we approach to the problems, questions and issues [2]. Learning clinical skills is an art and critical thinking is part and parcel to acquire this art.

Hyposkilliacs are “physicians who cannot take an adequate medical history, cannot perform a reliable physical examination, cannot critically assess the information they gather, cannot create a sound management plan, have little reasoning power, and communicate poorly. They learn to order all kinds of tests and procedures but don't always know when to order [them] or how to interpret them” [3]

The requisite of producing good clinicians is to create an environment of inquiry. The learner or clinician will analyze and evaluate the patient and disease with his/her cognitive skills only if his brain is primed to do so. Otherwise the culture of technological overuse will keep on flourishing; and we will end up in producing clinicians unable to pick up simple clinical diagnoses with poor basic knowledge and skills. This is high time to revisit the training methods for junior doctors, to be addressed conscientiously.

## Footnotes

**Source of Support:** Nil

**Conflict of Interest:** None declared
